# Importance of the Physical Exam in Diagnosing Ludwig’s Angina Without Access to Modern Imaging Modalities in the Developing World

**DOI:** 10.7759/cureus.74210

**Published:** 2024-11-22

**Authors:** Thaddeus Z Wegrzyn, Jacob S Greenberg

**Affiliations:** 1 Family and Community Medicine, Edward Via College of Osteopathic Medicine, Blacksburg, USA; 2 Internal Medicine, Johnston Memorial Hospital, Abingdon, USA

**Keywords:** airway emergency, ludwig's angina, oral infectious diseases, rural health settings, swelling of neck

## Abstract

Ludwig’s angina (LA) is a rapidly progressive cellulitis-causing airway obstruction that can spread through fascial planes to the floor of the mouth and into the mediastinum. Early recognition and treatment are essential for preventing potentially fatal complications. Diagnosis is based on clinical suspicion and confirmed through CT and ultrasound (US). In areas lacking access to imaging modalities, clinicians must rely on a thorough physical examination. Reporting this case is to raise awareness for LA in underserved areas with a higher risk for infection.

The case describes a 54-year-old female with a two-day history of an untreated throat infection that likely developed LA. She presented with a sore throat, progressively worsening shortness of breath, and submandibular swelling accompanied by self-reported fever, chills, and night sweats. With the limited resources available at the rural clinic, she was treated with oral metronidazole, amoxicillin, as well as prednisone. She was referred to the nearest hospital for an immediate surgical consultation.

## Introduction

Ludwig’s angina (LA) is a rapidly progressive cellulitis causing airway obstruction [1-3]. The infection begins in the submandibular space and can spread through fascial planes to the floor of the mouth and into the mediastinum, leading to high rates of fatality if untreated [1-2]. Because the roots of the lower molars extend into this space, infections of the molars can lead to LA [3].

Immunocompromised states and poor oral hygiene are well-documented risk factors for LA [2-3]. The most common etiology (90% of cases) of LA is dental infections, with *Streptococcus viridans* being identified as the most common causative pathogen [1-2]. Patients can present with tense swelling of the submandibular space, trismus, a “double-tongue” appearance, fever, “hot potato” voice, general malaise, and airway obstruction symptoms (i.e., stridor, tripod positioning, tachypnea) [1-4]. The infection may extend into the chest, causing mediastinitis [2,5]. LA can be highly suspected with the classic clinical exam findings and confirmed using CT or ultrasound (US) [3,4,6]. CT and US findings include thickening of the soft tissue, abscess, muscle edema, and loss of fat planes [3,6].

Broad-spectrum antibiotics, airway management, and surgical decompression are the standard methods of treatment [1-2]. Steroids have also been found to have a potential benefit in the management of LA, but their role is still being investigated [1]. Mortality of untreated LA can be as high as 50-60%, but with prompt diagnosis and treatment, it can be well under 10% [2-3].

## Case presentation

A 54-year-old female with a past medical history of dentures, hypertension, and gastritis presented to the Edward Via College of Osteopathic Medicine (VCOM) medical outreach clinic in Santa Rosa de Copan, Honduras, with complaints of a sore throat and progressively worsening shortness of breath, exacerbated by exertion over the past day. She reported going to the local hospital two days prior, where they told her she had pharyngitis. She was prescribed antibiotics but stated she was unable to afford them from the pharmacy. She reported pain with swallowing and constant, burning pain in her submandibular and sublingual areas that worsened with palpation. A review of systems revealed pain with swallowing and self-reported fever, chills, and night sweats. She also reported feeling dizzy when bending over for the past day. She endorsed shortness of breath and palpitations on exertion. Her current medications include irbesartan for hypertension and omeprazole for gastritis. Vital signs are listed in Table 1.

**Table 1:** Patient's vital signs

Vital signs	
Pulse	112 bpm
Blood pressure	138/90 mmHg
Respirations	28 breaths per minute
Temperature	98^o^ F (36.7^o^ C)

**Figure 1:**
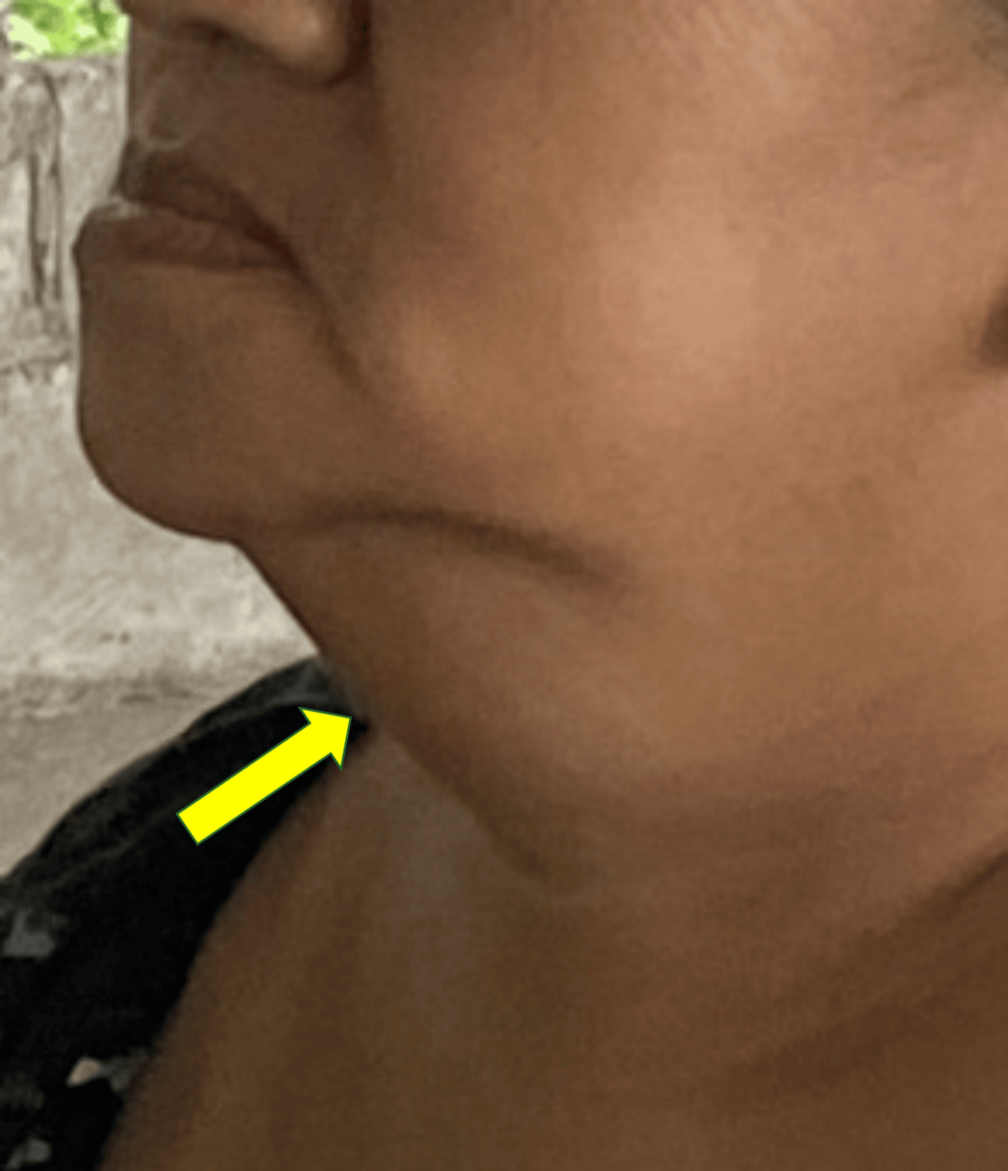
Submandibular swelling Clinical presentation of our patient demonstrating bilateral submandibular swelling. The swelling is firm without any erythema or external skin discoloration.

The patient was observed to be in the tripod position while seated on the exam table with shortness of breath on exertion and a “hot potato voice” when speaking. Swelling without erythema (Figure 1) was noted on inspection of her submandibular region and was firm on palpation with exquisite tenderness. Auscultation of the lungs was significant for inspiratory stridor. An oral exam revealed dentures with poor oral hygiene, and swelling of the floor of the mouth caused a "double tongue" appearance. The patient had difficulty opening her mouth all the way due to choking.

Without access to further imaging modalities, a preliminary diagnosis of LA was made based on the history and physical exam findings. With the limited resources available at our clinic, empiric antibiotic treatment of oral metronidazole and amoxicillin was initiated. Airway management included giving nebulized prednisone. The patient was then advised to go straight to the emergency department for further imaging and surgical evaluation. On follow-up of the patient months later, our patient reported that she had not followed instructions in the referral because she felt symptom relief following initial management. She continued to take the antibiotics given to her at the clinic until they were complete and afterward received regular penicillin injections for a few months from a different medical organization.

## Discussion

In the developed world, in addition to a thorough history and physical exam, diagnostic modalities, i.e., CT and US, are routinely used to diagnose LA [3,4]. In the developing world, where resources are scarce, prompt recognition and empiric treatment should be considered if there is a high suspicion of LA due to its high mortality rate if untreated [1,7].

Odontogenic infections are the most common cause of LA, and physicians should be aware if a patient presents with a recent history of dental infection or trauma. Rural populations, especially in developing countries, are at higher risk of odontogenic infections from poor oral hygiene, limited transportation and access to proper dental care, and a lack of preventative oral health measures. Lack of access further increases the risk of mortality due to the delay in seeking care [8-13]. Our patient had seen a local doctor regarding her throat infection two days prior but was unable to afford and start her antibiotics as prescribed.

This, in addition to her poor oral hygiene and history of dentures, may have predisposed her to the spread of infection that possibly developed into LA. Of note, our patient was afebrile upon presentation. While LA classically presents with a high fever, other case reports have reported similar findings [14-16].

Because of a thorough physical exam, we were able to educate the patient on the severity of her illness and act. LA has a high morbidity and mortality rate if untreated; further investigations into its prevalence in the developing world could be of utility. In addition, focusing on prevention by improving accessibility to affordable dentistry and frequent oral exams by local community providers might be of utility to decrease the incidence of LA.

## Conclusions

LA is a rare disease that can lead to severe complications and high mortality without early diagnosis and treatment. In underserved areas with minimal technology and a higher prevalence of infections, physicians must rely heavily on history and physical exams to make the diagnosis and educate the patient. Including LA in the differential diagnosis can ultimately prevent mortality in patients with treatable complications.
